# Testing Hepatitis E Seroprevalence among HIV-Infected Patients in Greece: The SHIP Study

**DOI:** 10.3390/pathogens13070536

**Published:** 2024-06-24

**Authors:** Nikolina Antonopoulou, Georgios Schinas, Zoi Kotsiri, Olga Tsachouridou, Konstantinos Protopapas, Vasileios Petrakis, Emmanouil C. Petrakis, Despoina Papageorgiou, Dimosthenis Tzimotoudis, Simeon Metallidis, Antonios Papadopoulos, Markos Marangos, Emmanouil Barbounakis, Diamantis P. Kofteridis, Periklis Panagopoulos, Charalambos Gogos, Apostolos Vantarakis, Karolina Akinosoglou

**Affiliations:** 1 Department of Internal Medicine and Infectious Diseases, University of Patras, 26504 Patras, Greece; nikolina.antonopoulou@gmail.com (N.A.); mmarangos@yahoo.com (M.M.); 2 School of Medicine, University of Patras, 26504 Patras, Greece; georg.schinas@gmail.com (G.S.); dspn.pap96@gmail.com (D.P.); cgogos@med.upatras.gr (C.G.); 3 Department of Public Health, University of Patras, 26504 Patras, Greece; zoikotsiri@gmail.com (Z.K.); dtzimotoudis@gmail.com (D.T.); avanta@med.upatras.gr (A.V.); 4 Department of Internal Medicine and Infectious Diseases AHEPA Hospital, Aristotle University of Thessaloniki, 54636 Thessaloniki, Greece; olgatsachouridou.iasis@gmail.com (O.T.); symeonam@auth.gr (S.M.); 5 4th Department of Internal Medicine, Medical School, National and Kapodistrian University of Athens, Attikon University General Hospital, 12462 Athens, Greece; kprotopapas@hotmail.com (K.P.); antipapa@med.uoa.gr (A.P.); 6 Department of Internal Medicine and Infectious Diseases, University General Hospital of Alexandroupolis, Democritus University of Thrace, 68100 Alexandroupolis, Greece; vasilispetrakis1994@gmail.com (V.P.); ppanago@med.duth.gr (P.P.); 7 Department of Internal Medicine and Infectious Diseases, University General Hospital of Heraklion, University of Crete, 71500 Heraklion, Greece; mpetrakis86@gmail.com (E.C.P.); barbouman2003@yahoo.gr (E.B.); kofterid@uoc.gr (D.P.K.)

**Keywords:** hepatitis E virus, HIV infections, coinfection, seroprevalence, Greece, antiretroviral therapy, highly active

## Abstract

Hepatitis E virus (HEV) poses significant health concerns worldwide, particularly among people living with HIV (PLWHIV), due to an increased risk of chronic infection and progression to cirrhosis in individuals with low CD4 cell counts. This study aimed to investigate the prevalence, chronicity potential, and risk factors of HEV infection among PLWHIV in Greece, where data are currently absent. A synchronic multicentric study encompassing five major Greek university hospitals was executed over 24 months, recruiting 696 PLWHIV participants. The prevalence of HEV IgG antibodies was 16.5%, with 8.6% showing evidence of acute HEV infection (HEV IgM). Active viral replication (HEV RNA) was present in 2.3% of the study population. Longitudinal analysis revealed that of the 25 initially anti-HEV IgM-positive individuals, only 3 seroconverted to IgG positivity, and among those with prior HEV RNA positivity (16), none showed evidence of active replication in subsequent tests. Comparative subgroup analysis highlighted the lack of significant differences in HIV-related parameters between HEV seropositive and seronegative individuals. Laboratory evaluations generally showed no significant disparities across most parameters; however, a higher seropositivity for Hepatitis A was observed in the HEV-positive subgroup. Our findings highlight a considerable prevalence of HEV among PLWHIV in Greece, with no observed cases of chronicity.

## 1. Introduction

Hepatitis E virus (HEV) is an enterically transmitted pathogen with a significant global footprint, causing major outbreaks and sporadic cases across various regions of the world. Despite its widespread prevalence and potential for severe complications, HEV remains under-acknowledged in public health dialogues. The disease typically follows a self-limiting course but also poses the grave risk of fulminant hepatic failure [1]. Moreover, immunocompromised hosts, including people living with HIV (PLWHIV) are at an increased risk of a chronic HEV infection trajectory, potentially accelerating the course to cirrhosis, especially in individuals with CD4 cell counts below 200 cells/µL [2,3]. Chronic HEV infection in this demographic can manifest with clinical presentations similar to opportunistic infections. Notably, clinical improvement may be achievable through immune restoration, highlighting the need for prompt and precise diagnosis to facilitate early intervention [2].

The co-existence of HEV with HIV complicates the clinical picture further, associating with a broad spectrum of extrahepatic manifestations. These include, but are not limited to, renal, hematological, pancreatic, and neurological disorders, significantly increasing morbidity and affecting the overall quality of life among infected individuals [4]. Recognizing and diagnosing co-infection early, particularly in cases of unexplained hypertransaminasemia or hepatic fibrosis in patients with diminished CD4 counts, is therefore of imminent importance. This allows for timely intervention to control viral replication and liver damage until antiretroviral therapy (ART) can facilitate immune reconstitution.

The immune response to HEV in PLWHIV diverges from the typical seroconversion pattern observed in the general population. Instead of a swift transition from IgM to sustained IgG antibodies conferring immunity, PLWHIV may exhibit prolonged HEV RNA presence without seroconversion, or only transient detection of IgG antibodies, indicating the establishment of a chronic infection state [5]. Data on the epidemiology of HEV and HIV co-infection remain sparse and inconsistent, reflecting variability across different studies and geographical settings [6,7,8,9,10,11]. This inconsistency underlines the urgent need for comprehensive research in this area.

In Greece, there has so far been no systematic screening for HEV among PLWHIV, unlike hepatitis B and C. The minimal data available stem from only two small studies conducted in 1996 and 2015, including 47 and 243 patients from a blood donation unit and a Greek specialized infections unit, respectively [12,13], which did not allow for reliable conclusions. Several questions remain unanswered, including whether the prevalence of anti-HEV IgG is the same among PLWHIV and the general population, whether PLWHIV constitute a high-risk group for HEV infection, and what factors influence and determine the transition to chronic infection (e.g., CD4 lymphocyte count).

Addressing these gaps, our study aims to elucidate the prevalence, potential for chronicity, and associated risk factors of HEV infection among PLWHIV in Greece, in order to contribute to the growing body of knowledge and help refine the clinical guidelines for managing and preventing this co-infection within a high-risk group.

## 2. Materials and Methods

### 2.1. Study Design

A synchronic multicentric study was designed to assess the prevalence of HEV infection and/or its chronicity among PLWHIV in Greece. Participating centers included five major university hospitals across the country, which serve as major referral centers for the care of PLWHIV, namely the following: (1) University General Hospital of Patras, (2) University General Hospital of Thesaloniki “AHEPA”, (3) University General Hospital of Heraclion, (4) University General Hospital of Alexandroupolis, and (5) University General Hospital of Athens “Attikon”. The study spanned a 24-month period, from January 2020 to December 2021, encompassing initial patient enrollment, blood sampling, and the collection of epidemiological data, followed by subsequent monitoring for possible rechecks, data analysis, and the dissemination of findings. 

The primary objective was to determine the prevalence and chronicity of HEV among PLWHIV in Greece. Secondary objectives were focused on evaluating factors potentially associated with HEV seroprevalence and/or chronicity and assessing hepatic injury parameters within the PLWHIV population. 

Conducted in adherence with Good Clinical Practice (GCP) guidelines and approved by the respective ethics and review boards (217/13-05-20), all patient data and samples were collected and stored anonymously, in line with General Data Protection Regulation (GDPR) requirements. Ethical considerations, particularly regarding the interpretation and communication of results to participants, especially for those diagnosed with chronic HEV infection, were carefully followed, ensuring that all participants were provided with appropriate counseling and medical advice.

### 2.2. Inclusion and Exclusion Criteria/Data Collection

Inclusion Criteria:

1. Adults (aged ≥ 18 years) diagnosed with HIV infection, as confirmed by standard diagnostic criteria, irrespective of the date of diagnosis or treatment.
2. Active follow-up at one of the participating centers.
3. Provision of written informed consent.
4. Available medical records for review of epidemiological, clinical, and laboratory data.

Exclusion Criteria:

1. Individuals younger than 18 years.
2. Patients who do not consent to participate in the study.
3. Insufficient medical records to assess HEV- and HIV-related clinical and laboratory parameters.
4. Inability to comply with study procedures, including follow-up visits and blood sample collections.
5. Concomitant participation in another interventional clinical trial that might interfere with the HEV study outcomes.

Data on epidemiological, socioeconomic, and behavioral parameters were collected from patient records, alongside extensive clinical and laboratory parameters. Additionally, the type and duration of the antiretroviral therapy were documented to evaluate its impact on HEV infection and chronicity. Diagnostic results for Hepatitis C virus (HCV), Hepatitis B virus (HBV), Hepatitis A virus (HAV) and other sexually transmitted diseases (STDs), obtained from the medical records, were derived from routine clinical assessments conducted according to established national and international clinical guidelines.

### 2.3. Sampling and Detection of Anti-HEV Antibodies and HEV RNA

Blood samples were collected, typically amounting to approximately 5 mL during a scheduled routine visit. Serum was isolated from these samples for the detection of anti- HEV IgM and IgG antibodies using an ELISA, Recomwell HEV Antibody kit (cat no: 5004 & 5005, Mikrogen, Neuried, Germany) according to the manufacturer’s instructions. The method shows sensitivity of 98.9% and specificity of 98.5%, and sensitivity of 98.9% and specificity of 98.6% for IgG and IgM, respectively. The final stage includes measuring the absorbance at 450 nm using a microplate reader (ChroMate-4300 ELISA reader, Awareness Technology, Inc., Palm City, FL, USA). In cases of positive anti-HEV IgM, molecular detection of HEV RNA was conducted. For this purpose, 200 µL of serum was used to isolate the viral RNA, utilizing a PureLink™ DNA/RNA mini kit (Catalog number: 12183018A, Thermo Fisher Scientific, Inc., Invitrogen, Carlsbad, CA, USA). Subsequently, RT-PCR was performed using a commercial kit Quanty^®^ HEV (code RT-41, Clonit srl, Milan, Italy), which was developed particularly to detect the ORF3 region of the HEV genome, following the manufacturer’s guidelines, in the thermocycler Stratagene Mx3005p (Agilent Technologies, Santa Clara, CA, USA) [14]. The cycling protocol was: initial denaturation at 50 °C for 30 min and 95 °C for 2 min; followed by 45 cycles of 95 °C for 15 s, annealing at 55 °C for 45 s and extension at 72 °C for 15 s. The technique demonstrates 100% clinical sensitivity and 100% diagnostic specificity. A follow-up sample was obtained after a minimum interval of 6 months to confirm or rule out chronic HEV co-infection.

### 2.4. Follow-Up Procedures

Follow-up visits were conducted at a minimum interval of 6 months to confirm or rule out chronic HEV co-infection. Criteria for determining the need for rechecks included RNA positivity and/or IgM positivity at the initial timepoint. During each follow-up, additional blood samples were collected to assess HEV RNA and antibody status, along with routine monitoring of liver function tests and CD4 counts.

### 2.5. Statistical Analysis

Continuous variables are presented as medians with interquartile ranges (IQRs), while categorical variables are summarized as frequencies and percentages. Statistical significance for comparisons among subgroups was determined using appropriate tests, based on the distribution of the data. The relationship between anti-HEV antibody presence and other variables was evaluated using a Chi-square and a Fisher’s exact test. All statistical analyses were carried out using SPSS software (version 28.0, IBM), and a *p*-value of <0.05 was considered statistically significant.

## 3. Results

Our multicentric study enrolled a cohort of 696 PLWHIV to assess the prevalence and potential chronicity of HEV infection. The demographic and clinical characteristics of the study population are summarized in Table 1A,B.

### 3.1. Population Characteristics

The median age of the participants was 46 years, with an interquartile range (IQR) of 39 to 55 years. A predominant majority (84.6%) were male. The median duration since HIV diagnosis was 9 years (IQR 5–14 years), and the median duration under antiretroviral therapy (ART) was 7 years (IQR 4–12 years). The majority of patients were asymptomatic (A1–A3, 66.8%), with smaller proportions in stages B1–B3 (14.3%) or bearing AIDS indicator conditions in stages C1–C3 (15.6%). Median HIV viral load was undetectable (0 copies/µL).

In terms of co-infections and other sexually transmitted diseases (STDs), 4.3% of participants were positive for Hepatitis B surface antigen (HBsAg), 7.4% had IgG antibodies against Hepatitis C Virus (HCV), and 22% had IgG antibodies against Hepatitis A Virus (HAV). Positive tests for syphilis and history of gonococcal disease were reported in 18.1% and 8.6% of the cohort, respectively.

### 3.2. HEV Screening Results

Out of the 696 participants, 16.5% were positive for HEV IgG antibodies, indicating exposure to HEV. Active HEV infection markers and HEV IgM antibodies were found in 8.6% of participants, with 5% showing concurrent positivity for both IgG and IgM antibodies. HEV RNA, confirming active viral replication, was detected in 2.3% of the population (Table 2). The median viral load for HEV among the patients who had a detectable load was 386 copies/mL (161–991 copies/mL). For the HEV IgG antibody titers, the quantification revealed a median of 35.03 IU/mL (28.82–55.81 IU/mL) for positive samples. For the HEV IgM antibody titers, the median was 31.62 IU/mL (26.66–42.31 IU/mL) for positive samples. 

**Figure 1:**
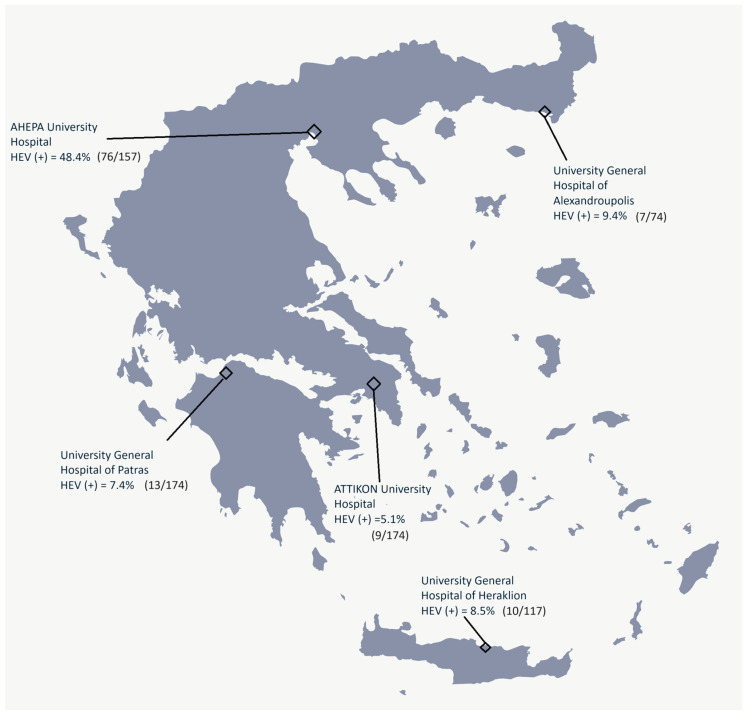
Geographic Distribution of Hepatitis E Virus (HEV) Seroprevalence in Greece.

Figure 1 summarizes the distribution of HEV screening results among participating centers in Greece.

### 3.3. Longitudinal Analysis

The repeated screening of participants with evidence of acute HEV infection (IgM positive/IgG negative) and those with prior HEV RNA positivity indicated a dynamic serological pattern. Of the 25 participants initially positive for anti-HEV IgM alone, 21 were retested, with only 3 demonstrating a seroconversion to IgG positivity. Importantly, none of those seroconverted was co-infected with HBV or HCV. 

Among those previously testing positive for HEV RNA, 2 were lost to the follow-up. Of the remaining 14, none showed evidence of active HEV replication in subsequent testing. As for their serological status, 3/4 previously tested IgG-negative had transitioned to IgG-positive. Notably, none of those seroconverted was co-infected with HBV or HCV.

### 3.4. Anti-HEV Positive Subgroup Analysis

The subgroup analysis compared the characteristics and laboratory parameters between HEV seropositive (IgG/IgM positive) [n = 140] and seronegative (IgG/IgM negative) [n = 556] individuals among people living with HIV (Table 3).

The proportion of male participants was comparable between the HEV-seropositive and seronegative groups (83.6% vs. 84.9%, *p* = 0.798). Median age and years since HIV diagnosis did not significantly differ between the two groups. Intravenous drug use (IVDU) was more prevalent in the HEV-seropositive group compared to the -seronegative group (11.6% vs. 6.1%, *p* = 0.042). Modes of HIV transmission, including sexual and bloodborne routes, showed no significant difference in prevalence between the two groups. Stages of HIV infection did not significantly vary between HEV-seropositive and seronegative individuals, either. CD4 cell counts were similar across both subgroups. Median HIV viral loads were undetectable in both subgroups.

The use of NRTIs, NNRTIs, INSTIs, and PIs was similar across both subgroups. A comparative analysis of co-infections revealed no significant differences in HBsAg and HCV antibody positivity. However, HAV antibody positivity was significantly higher in the HEV seropositive group (37.4% vs. 18.2%, *p* < 0.001). A past history of gonococcal disease was less common in the HEV-seropositive group (2.3% vs. 10.3%, *p* = 0.003). No participants in the HEV-seropositive group reported a history of chlamydia or other STDs, contrasting with the seronegative group (*p* < 0.001 and *p* = 0.002, respectively).

Laboratory evaluations showed no significant difference in white blood cell (WBC) count, platelet (PLT) count, serum glutamic oxaloacetic transaminase (SGOT), serum glutamic pyruvic transaminase (SGPT), urea, and creatinine levels. Notably, albumin levels were significantly higher in the HEV seropositive group (4.57 g/dL vs. 4.4 g/dL, *p* = 0.005). 

### 3.5. HEV-RNA-Positive and Anti-HEV IgM-Positive Subgroups’ Analysis

The following tables summarize the demographic, clinical, and laboratory characteristics of the subgroups of participants who were HEV RNA-positive and those who were anti-HEV IgM-positive (Table 4 and Table 5). The HEV-RNA-positive subgroup had a median age of 43 years and a median CD4 count of 673 cells/µL, with the majority of individuals being male (81.3%) and MSM (50%). In contrast, the anti-HEV IgM-positive subgroup, with a longer median duration of treatment (9 years), had a higher proportion of HAV Ab and HCV Ab positivity (41.6% vs. 18.9% and 13.3% vs. 6.3%, respectively) and a relatively better-preserved immune status as indicated by a higher median CD4 count of 779.5 cells/µL.

## 4. Discussion

This is the first study assessing HEV seroprevalence among PLWHIV in Greece. In our Greek nationwide cohort of 696 patients, we have shown an HEV seroprevalence of 16.5% among PLWHIV, while no cases of chronic infection were detected. Even though no differences among seropositive and seronegative patients were observed regarding HIV-associated parameters and laboratory liver function tests, a significant association of seropositivity with HAV was noted.

The immunological response following HEV infection typically follows a seroconversion pathway, beginning with a transient increase in IgM antibodies leading to a gradual and persistent presence of IgG antibodies, leaving behind partial immunity. However, in PLWHIV, HEV can persist and progress into a chronic infection, characterized by the presence of HEV RNA for more than 3 months. In these patients, the seroconversion from IgM to IgG may be absent, or the IgG may only be detectable transiently. Additionally, PLWHIV are known to be susceptible to re-infection with HEV. In such cases of re-infection, the immunological profile is marked by the presence of HEV RNA in serum with positive IgG antibodies and a consistent absence of IgM antibodies. As a result, the only marker capable of distinguishing between re-infection and chronic infection is the persistence of HEV RNA.

Our study findings reveal important insights into the immunological response and serological patterns of HEV infection in PLWHIV. The observed seroprevalence of HEV IgG antibodies (16.5%) indicates a significant exposure to HEV among this population. The detection of active HEV infection markers in 8.6% of participants, along with the presence of HEV RNA in 2.3%, suggests ongoing viral replication in a subset of individuals. These results highlight the dynamic nature of HEV infection in PLWHIV, where typical seroconversion patterns may be disrupted. Moreover, the low rate of seroconversion from IgM to IgG observed in our study (only 3 out of 25 initially IgM-positive participants) reveals the potential for chronic HEV infection in PLWHIV, despite the absence of chronic cases in our cohort. This finding suggests that the immune response in PLWHIV may be insufficient to clear the virus effectively, leading to prolonged HEV RNA presence without stable IgG seroconversion. Additionally, the fact that none of the participants who seroconverted were co-infected with HBV or HCV indicates that these co-infections might significantly impact the serological outcome of HEV infection in this context. Moreover, the absence of active HEV replication upon follow-up in previously RNA-positive individuals suggests a transient viremia in some PLWHIV, possibly due to intermittent viral clearance or suppression. This could also explain the normal liver enzyme levels observed, as low or fluctuating viral loads may not cause significant hepatic injury.

Epidemiological data on HIV/HEV co-infection are lacking, and findings from the few reports in the literature vary between studies and geographical regions. For instance, the seroprevalence of anti-HEV (IgG) among PLWHIV ranges from 1.1 to 16% in Europe [8,10,15,16], 7.1% to 45.3% in Africa [6,11,17], 2 to 9% in the USA [9,18], 44.2% in China [19], and 4.1 to 35.1% in South America [9,20,21]. The exact incidence of chronic HEV infection among PLWHIV appears to be rare, between 0 and 1%; however, corresponding data in global literature are exceedingly scarce and limited [3].

In HIV-infected individuals, there seems to be a greater likelihood of exposure to HEV compared to the general population. Nevertheless, the incidence of chronic HEV infection appears to be relatively minimal overall [22,23]. For instance, in a study involving 448 HIV-infected patients, anti-HEV IgG was detected in 10.4% of participants, but HEV RNA was only found in 1 out of 45 patients who were tested [23]. In addressing whether HIV infection actually poses a risk factor of HEV infection, recent efforts have aimed to clarify this relationship through comparative population-based analyses. The most recent meta-analysis on the matter, which aggregates data from a wide array of geographical locations and studies, posits that there is no significant difference in the prevalence of HEV among PLWHIV compared to the general population [24]. Moreover, a systematic review focused on Africa—a region noted for its higher overall prevalence of both HIV and HEV—mirrors the meta-analysis’s findings by also indicating no discernible difference in HEV prevalence between PLWHIV and the general population [25]. The concordance of these findings suggests that HIV infection per se does not inherently elevate the risk of acquiring HEV, at least in the broad context analyzed.

In assessing the prevalence of HEV within the general population, it is important to acknowledge the variability of infection rates and potential risk factors across global regions, along with the inherent heterogeneity of study protocols utilized to determine seroprevalence. These include analyses of healthy blood donor banks, individuals with acute hepatitis, and broader general population studies. A recent review, informed by data from the Balkan region, indicates that the HEV-3 genotype predominates in this area of the world, suggesting zoonotic transmission with wild animal hunting identified as a primary infection risk factor [26]. The review reports an anti-HEV IgG seroprevalence rate in Greece of up to 9.7%, derived from a study concentrating on blood donors with a relatively modest sample size of 265 individuals [27]. In comparison, neighboring countries report a wider range of prevalence: 1.1–24.5% in Croatia, up to 20.9% in Bulgaria, 5.9–17.1% in Romania, 15% in Serbia, and 2–9.7% in Albania. Findings from a subsequent, relatively large blood donor study in Bulgaria, set the bar even higher as the authors identified anti-HEV IgG positivity in 25.9% of participants, while reinforcing wild animal hunting as a significant infection risk factor [28]. As for HEV RNA detection among the general population, a comprehensive study from Croatia revealed a low prevalence rate, identifying HEV RNA in only four out of 8631 blood donations tested, which corresponds to an incidence rate of one in 2158 donations or 0.046%, aligning with recent European epidemiological data [29].

Risk factors for HEV infection among PLWHIV vary by region and individual behaviors, while traditional risk factors for HEV exposure (e.g., consumption of contaminated water or undercooked meat) should not go unnoticed. For instance, a study conducted in Brazil identified piped water availability as a protective factor against HEV infection, suggesting that lack of access to clean water is a risk factor [20]. Specific risk behaviors that are more prevalent among the HIV demographic, such as men who have sex with men (MSM), might also influence the risk of HEV infection. Reportedly, MSM have two-fold increased odds of HEV infection according to a study from Italy [30]. This comes in accordance with increased prevalence of HAV seropositivity in this subgroup of patients, reflecting similar ways of transmission including sexual intercourse practices [31]. Interestingly, in our cohort, MSM proportions were not significantly different across HEV subgroups.

The geographic distribution of observed HEV cases reveals a pronounced gradient in HEV seroprevalence across Greece. Notably, the northern regions, for which the AHEPA University Hospital of Thessaloniki serves as a reference center, exhibit a seroprevalence rate of 48.4%. This contrast is substantial when compared to the lower prevalence rates noted in the southern regions, such as the 7.4% at the University General Hospital of Patras and the 5.1% at the ATTIKON University Hospital of Athens. This observation conflicts with prior reports indicating increased rates in southern parts of Greece, particularly among male blood donors in Crete (13.3%) [32]. Data deriving from the urban population in Athens have demonstrated a seroprevalence of 7.3–9.43% [12,27]. Studies from Thesallia among health donors also showed a varying seroprevalence of 1.8–9.8% [33]. In Epirus and the Agrinion region, seropositivity was much lower reaching 5.3% among patients with chronic viral hepatitis, 5.2% among open heart surgical patients, 1.34% in patients on hemodialysis, and barely reaching 0.23% among healthy blood donors [34,35]. A recent study in northern Greece confirmed no past or acute HEV infection in transfusion-dependent thalassemia patients [36], while a recent study in the same area found that only 1.3% of liver transplant patients were positive for HEV RNA [37].

The exact reasons for the increased seroprevalence in northern Greece remain to be explored, although local outbreaks remain a possibility. Increased seropositivity in our study could be partly explained by the proximity and variable population movement from/to countries where the disease is endemic, including countries of southeastern Europe and Asia, since the north and northeastern parts of Greece represent the main points of entry from these areas [26,38,39]. This significant within-country variability possibly reflects variable eating habits and cultural or religious norms, particularly among diverse groups inhabiting northeastern areas, e.g., Pomaks. Moreover, such a disparity suggests potential regional variations in risk factors, which may include differences in environmental exposures, dietary habits, agricultural practices, or the presence of zoonotic reservoirs. A recent study in a leafy green vegetable supply chain in Greece showed that HEV RNA was found in 4.76% and 3.2% of samples from the primary production and point-of-sale phases, respectively [40]. Similarly, the presence of HEV in irrigation water samples used for the production of leafy green vegetables showed that 1 out of 20 samples tested positive [41]. Further research is pivotal, especially in environmental samples and animals in individual regions in Greece to gain a better insight into local HEV epidemiology.

Finally, research has begun to explore the role of genetic factors in susceptibility to HEV infection among PLWHIV. For example, a mutation in the progesterone receptor has been associated with predisposition to HEV infection in PLWHIV [42]. As for heightened chronic HEV risk, the level of immunosuppression in PLWHIV may significantly influence the course of the infection. Lower CD4 counts may affect the progression to chronic HEV infection, especially in those not receiving effective ART [17]. Conversely, effective ART leading to viral suppression and immune reconstitution could potentially prevent its progression to chronicity, as in the case of HBV/HIV co-infection [43]. Additionally, PLWHIV are at an increased risk of coinfections with other hepatitis viruses, such as HBV and hepatitis C virus (HCV), due to shared routes of transmission. Such coinfections can influence the trajectory of HEV infection, affecting clinical outcomes and thereby necessitating comprehensive management strategies [44]. Notably, within our study cohort, the prevalence of HCV and HBV antibody positivity was nearly twice as high even though not significant among those infected with HEV, highlighting the increased morbidity burden within this subgroup. This comorbidity status, along with the pre-existing hepatic-specific involvement, may have contributed to their susceptibility to HEV and/or resulted in more severe presentations. Conversely, extrahepatic infections, such as STDs, were significantly more prevalent in the non-HEV-infected subgroup.

Our findings highlight the importance of following current guidelines for HEV testing among PLWHIV, as recommended by EACS 2023 and EASL 2022 [5,45]. These guidelines emphasize the need for prioritizing HEV testing in individuals showing symptoms of acute hepatitis, unexplained liver enzyme elevations, or extrahepatic manifestations such as amyotrophy, Guillain-Barré syndrome, encephalitis, or proteinuria. Recommended tests include anti-HEV IgG and IgM, as well as NAAT for HEV RNA in the blood and, if possible, in the stool. It is critical to consider HEV infection in PLWHIV, even when initial serological tests are negative, especially in those with low CD4 cell counts. PCR testing for HEV RNA should be performed to ensure accurate diagnosis. Additionally, routine screening for HEV should be a priority for PLWHIV with pre-existing liver conditions, as HEV infection can become severe in these cases. For this group, follow-up testing is essential to confirm that an initial infection does not progress to chronicity. Failing to implement these guidelines can lead to underestimating the disease, as HEV infections may often go undetected due to spontaneous clearance after ART initiation. A recent ECDC report pointed out the inconsistent implementation of routine HEV testing and surveillance across EU/EEA member states [46]. This inconsistency has prompted the WHO to stress the importance of strengthening national laboratory systems to ensure the availability of diagnostics and high-quality diagnosis for both acute and chronic HEV infections [47].

Our report bears a number of limitations that limit our understanding of the natural history of HEV infection in PLWHIV. Even though this was a nationwide study including a representative population from HIV referral centers across the country, the sample was limited to populations with good access and engagement to care. It remains unknown whether difficult-to-treat populations, including moving populations, minorities, and IVDU, exhibit the same seroprevalence. Τhe country of origin of the participants was also not disclosed in our study. While our current dataset included a longitudinal follow-up of a subset of patients, expanding this to the entire cohort was impossible due to loss to follow-up. Additionally, our epidemiologic data collection remained largely retrospective and reliant on patient records, hence posing risks for reporting bias and incomplete information. Moreover, specific risk factors such as dietary habits and the use of bottled water were not available for our cohort at the time of data collection. These factors were not included in the initial study design, which focused primarily on clinical and serological parameters. A broader epidemiological approach is recommended for future studies to assess potential exposure routes more comprehensively.

Furthermore, a number of unresolved issues persist, including the duration of seropositivity and the exact impact of immune reconstitution and ART on infection resolution. Additionally, genomic sequencing of HEV strains was not feasible within the constraints of this study. Such data could elucidate the types of circulating viruses, particularly distinguishing between autochthonous and imported infections. Therefore, genomic sequencing is recommended for future research to potentially identify different genotypic impacts on the disease course among PLWHIV. Finally, the measurement unit (U/mL) used in the Mikrogen kit is arbitrary, which does not allow for direct comparison with international reference values. As a result, we are unable to correlate our findings with established protective thresholds, such as the 7 U/mL threshold noted to be protective against reinfection in solid organ transplant patients and rhesus monkeys [48,49].

## 5. Conclusions

To conclude, our report showed a 16.5% prevalence of HEV among PLWHIV in Greece, although no cases of chronicity were observed. Findings vary significantly across regions, suggesting that the seroprevalence of HEV in Greece, as well as potentially in other neighboring countries, may have been underestimated. Factors contributing to this underestimation may include demographic and geographic diversity of the sampled populations, differences in study methodologies, and small sample sizes. Future research should focus on understanding the dynamics of HEV infection in this population, exploring potential protective factors, and developing effective prevention and management strategies. Considering the critical need for accurate prevalence estimates for effective public health planning, our findings highlight the necessity for larger and more representative samples, along with comprehensive genomic screening, to ascertain the true prevalence of HEV in Greece and across the broader region more accurately.

## Figures and Tables

**Table 1:** (**A**) Population characteristics (n = 696). (**B**) HIV-related information of the population (n = 696).

**(A)**	
Age (IQR)	46 (39–55)
Male sex (%)	589 (84.6%)
Years since Dx (IQR)	9 (5–14)
Years under Tx (IQR)	7 (4–12)
CD4 cell count (cell/µL)	687 (491–910)
**(B)**	
Risk Factors	
Intravenous drug use (IVDU) (%)	50 (7.2%)
MSM (%)	316 (45.4%)
Mode of HIV transmission	
Blood (%)	42 (6%)
Sex (%)	421 (60.5%)
Unknown (%)	233 (33.5%)
ART regimen included:	
NRTI (%)	551 (79.2%)
NNRTI (%)	117 (16.8%)
INSTI (%)	452 (64.9%)
PI (%)	136 (19.5%)
HIV Stage at time of enrollment:	
A1–A3 (%)	465 (66.8%)
B1–B3 (%)	100 (14.3%)
C1–C3 (%)	109 (15.6%)
Unknown (%)	20 (2.8%)
Infectious disease panel	
HBsAg positive (%)	30 (4.3%)
Anti-HCV IgG positive (%)	51 (7.3%)
Anti-HAV IgG positive (%)	152 (21.8%)
Syphilis test positive (%)	124 (17.8%)
History of tuberculosis (TB) (%)	12 (1.7%)
History of gonococcal disease (%)	55 (7.9%)
History of chlamydia (%)	39 (5.6%)
Other STDs (%)	29 (4.2%)

IQR: Interquartile Range, n: Number, Dx: Diagnosis, Tx: Treatment, HIV: Human Immunodeficiency Virus, CD4: Cluster of Differentiation 4, IVDU: Intravenous Drug Use, MSM: Men who have sex with men, HEV: Hepatitis E Virus, ART: Antiretroviral Therapy, NRTI: Nucleoside Reverse Transcriptase Inhibitor, NNRTI: Non-Nucleoside Reverse Transcriptase Inhibitor, INSTI: Integrase Strand Transfer Inhibitor, PI: Protease Inhibitor, HBsAg: Hepatitis B Surface Antigen, HCV: Hepatitis C Virus, HAV: Hepatitis A Virus, TB: Tuberculosis, STDs: Sexually Transmitted Diseases.

**Table 2:** HEV Screening in Greece (n = 696).

HEV IgG positive (%)	115 (16.5%)
HEV IgM positive (%)	60 (8.6%)
HEV IgM positive/IgG positive (%)	35 (5%)
HEV IgM positive/IgG negative (%)	25 (3.6%)
HEV IgG positive/IgM negative (%)	80 (11.5%)
HEV IgM negative/IgG negative (%)	516 (74.1%)
HEV RNA positive (%)	16 (2.3%)

**Table 3:** Comparative Analysis Between HEV (+) and (−) Subgroups.

	HEV IgG/IgM (+) [n = 140]	HEV IgG/IgM (−) [n = 556]	*p*-Value
Male sex (%)	117 (83.6%)	472 (84.9%)	0.798
Age (IQR)	49 (42–57)	46 (39–54)	0.156
Years since Dx (IQR)	9 (5–14)	8 (4–13)	0.830
Years under Tx (IQR)	7 (4–11)	8 (4–12)	0.877
IVDU (%)	16 (11.6%)	34 (6.1%)	0.042
MSM (%)	58 (42.3%)	258 (46.7%)	0.407
Bloodborne transmission (%)	13 (9.4%)	29 (5.2%)	0.100
Sexual transmission (%)	80 (58%)	341 (61.5%)	0.500
NRTI (%)	112 (80%)	439 (79%)	0.243
NNRTI (%)	19 (13.6%)	98 (17.6%)	0.193
INSTI (%)	88 (62.9%)	364 (65.5%)	0.066
PI (%)	33 (23.6%)	103 (18.5%)	0.461
HBsAg (%)	10 (7.2%)	20 (3.6%)	0.106
HCV Ab (%)	16 (11.6%)	35 (6.3%)	0.056
HAV Ab (%)	52 (37.1%)	100 (18%)	<0.001
RPR/VDRL/TPHA (+) (%)	31 (22.1%)	93 (17%)	0.160
TB (%)	3 (2.1%)	9 (1.6%)	0.714
Past history of gonococcal disease (%)	3 (2.1%)	52 (9.3%)	0.003
Past history of chlamydia (%)	0 (0%)	39 (7%)	<0.001
Other STDs (%)	0 (0%)	29 (5.2%)	0.002
HIV Stage			
A1–A3 (%)	85 (60.7%)	380 (68.4%)	0.089
B1–B3 (%)	26 (18.6%)	74 (13.3%)	0.137
C1–C3 (%)	26 (18.6%)	83 (14.9%)	0.299
Unknown Stage (%)	3 (2.1%)	17 (3%)	0.779
Laboratory Value			
WBC (×10^3^/uL)	6570 (4960–8180)	6440 (5090–7790)	0.766
PLT (×10^3^/uL)	253 (217–289)	239 (197–281)	0.140
SGOT (U/L)	22 (16–28)	22 (17–27)	0.307
SGPT (U/L)	24 (15.5–32.5)	24 (15.5–32.5)	0.863
Albumin (g/dL)	4.57 (4.24–4.78)	4.4 (4.1–4.7)	0.01
Globulin (g/dL)	3.1 (2.97–3.67)	3 (2.7–3.3)	0.049
Urea (mg/dL)	31 (25.5–36.5)	32 (26.4–37.6)	0.102
Creatinine (mg/dL)	0.95 (0.81–1.09)	1.0 (0.87–1.14)	0.212
CD4 Cell Count	672 (488–896)	692(492–919)	0.964

IQR: Interquartile Range, IVDU: Intravenous Drug Use, MSM: Men who have sex with men, HEV: Hepatitis E Virus, n: Number, Dx: Diagnosis, Tx: Treatment, HIV: Human Immunodeficiency Virus, ART: Antiretroviral Therapy, CD4: Cluster of Differentiation 4, NRTI: Nucleoside Reverse Transcriptase Inhibitor, NNRTI: Non-Nucleoside Reverse Transcriptase Inhibitor, INSTI: Integrase Strand Transfer Inhibitor, PI: Protease Inhibitor, HBsAg: Hepatitis B Surface Antigen, HCV: Hepatitis C Virus, HAV: Hepatitis A Virus, RPR/VDRL: Rapid Plasma Reagin/Venereal Disease Research Laboratory, TB: Tuberculosis, STDs: Sexually Transmitted Diseases, HEV Virological information, IgM: Immunoglobulin M, WBC: White Blood Cell count, PLT: Platelet count, SGOT: Serum Glutamic Oxaloacetic Transaminase, SGPT: Serum Glutamic Pyruvic Transaminase, Alb: Albumin, Glob: Globulin, Urea: Blood Urea Nitrogen, Cr: Creatinine.

**Table 4:** Characteristics of HEV-RNA-positive Subgroup (n = 16).

Characteristic	Value (IQR/%)
Age (years)	43 (33–59)
Male sex	13 (81.3%)
Years under Treatment	7 (5–8)
MSM	8 (50%)
IVDU	2 (12.5%)
Sexual transmission	8 (50%)
Bloodborne transmission	1 (6.3%)
HBsAg-positive	0 (0%)
HAV Ab-positive	3 (18.9%)
HCV Ab-positive	1 (6.3%)
Syphilis test-positive	3 (18.9%)
CD4 Cell Count (cells/µL)	673 (447–899)
SGOT (U/L)	28 (20–32)
SGPT (U/L)	26 (19–32)
Albumin (g/dL)	4.15 (3.9–4.6)

HEV: Hepatitis E virus, IQR: Interquartile Range, MSM: Men who have sex with men, IVDU: Intravenous drug user, HBsAg: Hepatitis B surface antigen, HAV: Hepatitis A virus, HCV: Hepatitis C virus, SGOT: Serum glutamic-oxaloacetic transaminase (AST), SGPT: Serum glutamic-pyruvic transaminase (ALT), g/dL: Grams per deciliter, µL: Microliter, U/L: Units per liter.

**Table 5:** Characteristics of Anti-HEV IgM-positive Subgroup (n = 60).

Characteristic	Value (IQR/%)
Age (years)	49 (43–56)
Male sex	50 (83.3%)
Years under Treatment	9 (6–14)
MSM	25 (41.6%)
IVDU	6 (10%)
Sexual transmission	29 (48.3%)
Bloodborne transmission	2 (3.3%)
HBsAg-positive	4 (6.6%)
HAV Ab-positive	25 (41.6%)
HCV Ab-positive	8 (13.3%)
Syphilis test-positive	10 (16.6%)
CD4 Cell Count (cells/µL)	779.5 (602–957)
SGOT (U/L)	21 (18–29)
SGPT (U/L)	23 (17–32)
Albumin (g/dL)	4.57 (4.26–4.83)

HEV: Hepatitis E virus, IgM: Immunoglobulin M, IQR: Interquartile Range, MSM: Men who have sex with men, IVDU: Intravenous drug user, HBsAg: Hepatitis B surface antigen, HAV: Hepatitis A virus, HCV: Hepatitis C virus, SGOT: Serum glutamic-oxaloacetic transaminase (AST), SGPT: Serum glutamic-pyruvic transaminase (ALT), g/dL: Grams per deciliter, µL: Microliter, U/L: Units per liter.

## Data Availability

Data are available upon reasonable request to the corresponding author.
